# Polyethersulfone (PES) Filters Improve the Recovery of *Legionella* spp. and Enhance Selectivity against Interfering Microorganisms in Water Samples

**DOI:** 10.3390/polym15122670

**Published:** 2023-06-13

**Authors:** Pablo Casino, Asunción López, Sara Peiró, Santiago Rios, Aldous Porta, Gemma Agustí, Daniela Terlevich, Daniel Asensio, Ana María Marqués, Núria Piqué

**Affiliations:** 1 Department of Quality Control, Reactivos para Diagnóstico, S.L. (RPD), Josep Tura, 9H, Polígon Industrial Mas d’en Cisa, Sentmenat, 08181 Barcelona, Catalonia, Spain; pacasinoalcalde45@gmail.com (P.C.); dtlab@reactivosparadiagnostico.com (A.L.); production@reactivosparadiagnostico.com (A.P.);; 2 Microbiology Section, Department of Biology, Healthcare and Environment, Faculty of Pharmacy and Food Sciences, Universitat de Barcelona (UB), Av. Joan XXIII, 27-31, 08028 Barcelona, Catalonia, Spain; 3 Department of Statistics, Biology Faculty, Universitat de Barcelona, Av. Diagonal, 643, 08028 Barcelona, Catalonia, Spain; 4 Institut de Recerca en Nutrició i Seguretat Alimentària de la UB (INSA-UB), Universitat de Barcelona, 08921 Barcelona, Catalonia, Spain

**Keywords:** *Legionella pneumophila*, *Legionella anisa*, GVPC, recovery, selectivity, *Pseudomonas aeruginosa*, *Enterococcus faecalis*, polyethersulfone (PES) filters

## Abstract

In the analysis of water samples, the type of filtration membrane material can influence the recovery of *Legionella* species, although this issue has been poorly investigated. Filtration membranes (0.45 µm) from different materials and manufacturers (numbered as 1, 2, 3, 4, and 5) were compared: mixed cellulose esters (MCEs), nitrocellulose (NC), and polyethersulfone (PES). After membrane filtration of samples, filters were placed directly onto GVPC agar and incubated at 36 ± 2 °C. The highest mean counts of colony-forming units and colony sizes for *Legionella pneumophila* and *Legionella anisa* were obtained with PES filters (*p* < 0.001). All membranes placed on GVPC agar totally inhibited *Escherichia coli* and *Enterococcus faecalis* ATCC 19443 and ATCC 29212, whereas only the PES filter from manufacturer 3 (3-PES) totally inhibited *Pseudomonas aeruginosa*. PES membrane performance also differed according to the manufacturer, with 3-PES providing the best productivity and selectivity. In real water samples, 3-PES also produced a higher *Legionella* recovery and better inhibition of interfering microorganisms. These results support the use of PES membranes in methods where the filter is placed directly on the culture media and not only in procedures where membrane filtration is followed by a washing step (according to ISO 11731:2017).

## 1. Introduction

Despite advances in our understanding of *Legionella* transmission and the use of improved methods to monitor *Legionella* in water samples and prevent the associated health risks [1,2], legionellosis remains one of the most frequent waterborne diseases [1,2,3,4,5]. Legionnaires’ disease, the pneumonic form of legionellosis, is typically caused by the inhalation of contaminated aerosolized water particles associated with warm water plumbing systems (e.g., evaporative cooling towers, hot- and cold-water distribution systems in buildings, and associated equipment such as spa pools) in which favorable temperatures (20–45 °C, optimum temperature of 37 °C) can foster *Legionella* growth [6,7]. Other important factors in *Legionella* colonization are an ability to survive and grow in biofilms and the presence of parasitizing amoebas [8].

The standard method for *Legionella* detection and enumeration in water samples is based on culture and membrane filtration [3,9]. The accuracy of *Legionella* enumeration in water needs to be improved for the correct validation of control measures and to assess the effectiveness of disinfection interventions [3,10]. In a recent study, modifications in the manufacture of the selective medium Glycine–Vancomycin–Polymyxin–Cycloheximide (GVPC) agar resulted in improved growth of *Legionella pneumophila* and *Legionella anisa* and a marked inhibition of *Escherichia coli*, *Pseudomonas aeruginosa*, and *Enterococcus faecalis* [10].

Another requiring improvement is the process of membrane filtration. ISO 11731:2017 stipulates that, due to the complex nature of sample matrices, the most appropriate sampling method should be selected by each laboratory. Concentration by membrane filtration (0.2 or 0.45 µm pore size) is usually required, particularly when levels of interfering microorganisms and *Legionella* are expected to be low [9]. To reduce the growth of non-target bacteria, which can interfere with the recovery of *Legionellae*, portions of water samples are also subjected to heat treatment, acid treatment, or a combination of both [9].

In procedures involving membrane filtration and direct placing of the membrane filter on culture media, filters of nitrocellulose (NC) or mixed cellulose esters (MCEs) are recommended, whereas when membrane filtration is followed by a washing procedure, filters of polycarbonate or polyethersulfone (PES) are considered more suitable. In the latter case, the microorganisms from the membrane filter are washed with sterile diluents using a vortex mixer or an ultrasonic water bath [9].

Despite these recommendations, there are still discrepancies about which filter provides the best recovery of *Legionella* spp. when the concentration of interfering microorganisms is low, and few comparative studies have been published to date. In a recent study by De Giglio et al., membranes with a pore size of 0.45 µm resulted in a greater recovery of *Legionella* than those of 0.2 µm, suggesting that the behavior of *Legionella* was related to bacterial interaction with the membrane rather than cell size [3]. It would therefore seem that the type of membrane can influence the recovery of *Legionella* and interfering microorganisms [3]. Different mechanisms could be involved, such as bacterial adhesion by electrostatic forces, diffusion of culture medium nutrients, and pore occlusion [3].

In a previous comparative study, different filters composed of various materials and pore sizes were evaluated. The filters providing the highest percentages of recovery were made of polycarbonate, although the results achieved with other materials, including PES, MCEs, cellulose acetate, NC, polyvinylidene difluoride, nylon, and ceramic, were not significantly different [11].

The material PES is currently attracting attention due to its excellent membrane formability and spinnability and hydrophobic properties, with applications in wastewater treatments (ultrafiltration) [12] and dialysis [13,14]. In *Legionella* testing, although PES filters are only recommended when membrane filtration is followed by a washing procedure [9], manufacturers point out that PES filters favor *Legionella* growth, suggesting their use could be extended to methods where the filter is placed directly on the culture media.

In this context, the present study was designed to test a range of filters of different materials and brands for the recovery of *Legionella* spp. from inoculated and real water samples containing interfering microorganisms. After sample filtration, the membrane filter was placed directly on the culture media. For the selective media, GVPC agar with improved productivity and selectivity properties was used, as previously described [10].

## 2. Material and Methods

### 2.1. Membrane Filters

The membranes used in this study to improve *Legionella* spp. recovery had a pore size of 0.45 µm [15] and were obtained from different manufacturers (Table 1). The materials and brands analyzed were as follows: two MCE membranes from brand 1 (white: 1-MCEw and black: 1-MCEb) and one membrane from brand 2 (2-MCE); one NC membrane from brand 3 (3-NC) and one from brand 4 (4-NC); and three PES membranes from different brands (3-PES, 2-PES, and 5-PES). NC filters from brand 4 (4-NC) were used as reference membranes since they are commonly used for *Legionella* recovery at the quality control department of the laboratory (Reactivos para Diagnóstico, S.L).

### 2.2. Legionella Inoculum Preparation

*L. pneumophila* (ATCC 33152; WDCM 00107) and *L. anisa* (ATCC 35292; WDCM 00106) were used according to ISO 11731:2017 [9].

Inocula were prepared according to ISO 11133:2014/amended 1:2018 [16]. The tested bacterial strains were obtained directly from a reference culture collection (American Type Culture Collection, ATCC). A single subculture from the reference strains was used to obtain reference stock strains from which stock and working cultures were prepared using the *Legionella* non-selective medium BCYE agar. Before use, each strain was verified in the appropriate selective and differential medium (GVPC agar) according to ISO 17025: 2017 [17].

Stock dilutions were prepared according to ISO 11731:2017 [9] and ISO 8199:2018 [18] and adjusted using turbidimetry (McFarland unit of 0.5 for productivity assays) (densitometer DEN-1B, Grant Instruments, Cambridgeshire, UK), from which serial dilutions were prepared in the same diluent. Serial dilutions were prepared to obtain 50–80 CFU per plate.

Samples of 100 µL containing <80 CFU of *Legionella* spp. were added to 50 mL of buffered peptone water 0.1% and then filtered through 0.45 µm mixed membrane filters. Filters were placed directly onto the GVPC medium with disinfected forceps, according to ISO 8199:2018 [18].

The plates were incubated at 36 ± 2 °C for 3 days for *L. pneumophila* and 5 days for *L. anisa*. Colony number and size were determined at days 3 and 5, respectively.

Colonies of *Legionella* were generally white–gray, although other colors could appear. Colonies were smooth with an entire edge and had a characteristic ground-glass appearance.

### 2.3. Selectivity Assays

Selectivity assays were performed according to ISO 11731:2017 [9] and ISO 11133:2014/AMD 1: 2018 (ISO 11133:2014/AMD 1:2018) [16] with the following strains: *E. faecalis* (ATCC 19433, equivalent to WDCM 00009 and ATCC 29212, equivalent to WDCM 00087) and *E. coli* (ATCC 25922, equivalent to WDCM 00013). *P. aeruginosa* (ATCC 9027, equivalent to WDCM 00026) was also tested.

Stock dilutions were adjusted using turbidimetry (McFarland unit of 0.5) (densitometer DEN-1B, Grant Instruments, Cambridgeshire, UK), from which serial dilutions were prepared in the same diluent.

Samples of 100 µL containing ≥10^3^ CFU of the strains were added to 50 mL of sterile water and then filtered through 0.45 µm membrane filters made of different materials. The filters were placed directly onto the GVPC medium with disinfected forceps, according to ISO 8199:2018 [18].

The plates were incubated at 36 ± 2 °C for three-day periods, after which the CFU count was obtained for all strains.

### 2.4. Solid Media

GVPC medium used to assess the membranes was manufactured by Reactivos para Diagnóstico S.L. (Sentmenat, Barcelona, Spain), as previously described (autoclaving at 115 °C for 15 min and ingredient mixing without oxygen) [10].

### 2.5. Colony Count

The colony count was performed manually. The size of colonies was obtained first in pixels and then converted to mm using the mobile application Pixel Measure 1.0 (Leroy Hopson Apps, Vietnam).

### 2.6. Analysis of Real Water Samples

Real water samples were provided by Aconsa, S.L. (Barcelona, Spain): eight water samples with a low concentration of interfering microorganisms (LIM) and five water samples with a high concentration of interfering microorganisms (HIM). Filters of different materials (polyethersulfone, mixed cellulose esters, and nitrocellulose) and brands were used.

LIM and HIM water samples of 1 L were collected aseptically in sterile containers containing a neutralizing agent and transported to the laboratory, where they were stored at 5 ± 3 °C until needed.

For each LIM water sample, two aliquots were analyzed: direct samples and laboratory samples contaminated with *L. pneumophila* (500–1000 CFU/L) and *L. anisa* (500–1000 CFU/L). Different volumes of sample were filtered (1, 10, and 100 mL) and plated onto GVPC agar.

For each HIM water sample, three 100 mL aliquots were prepared. Each aliquot was contaminated with *L. pneumophila* (500–1000 CFU/L) and *L. anisa* (500–1000 CFU/L) strains and submitted to different conditions: (a) water without pre-treatment; (b) water pre-treated thermically (50 °C for 30 min), and (c) water with acid buffer added (HCl-ClK buffer, pH 2.2 for 5 min). The aliquots were filtered and plated onto GVPC agar.

The concentration of the added *L. pneumophila* and *L. anisa* strains was variable, depending on the analyzed aliquot: the untreated and thermically pre-treated aliquots were contaminated with 500–1000 CFU/L of *L. pneumophila* and 500–1000 CFU/L of *L. anisa*, while the aliquots pre-treated with acid buffer were inoculated with concentrations 10-fold higher (5000–10,000 CFU/L per strain).

The plates were incubated at 36 ± 2 °C for four days and were inspected for the first time on day 2.

### 2.7. Statistical Analysis

The initial experiments with membranes composed of different materials were performed in triplicate and repeated on five different days. All data were analyzed by a general linear model using SPSS v.21.0 (IBM Corp., Chicago, IL, USA). The means and standard deviations were calculated for all measures (number of colonies/plates, size of colonies). The numbers of CFU obtained in the media using different membrane filters were subjected to an analysis of variance (ANOVA test) using the general linear model procedure to eliminate inter-day variability. A post hoc analysis using Fisher’s least significant difference test was performed to compare the filters individually and identify the differences between them. The number of bacterial colonies growing on an agar plate was presumed to follow a Poisson distribution, so the square root was extracted to normalize the data and to apply the ANOVA tests. The CFU counts obtained from the same suspension and the size of colonies (mm) grown using different membranes were compared using the Student’s *t*-test for independent data.

For the comparison of PES filters, five repetitions were performed on the same day and using the same inoculum. The means and standard deviations were calculated for all measures (number of colonies/plates, size of colonies) and compared using the Student’s *t*-test for independent data. In all tests, the significance level alpha was set as 0.05.

### 2.8. 16S Sequencing of Water Samples

LIM and HIM water samples were aliquoted individually in 1 mL Eppendorfs and centrifuged at 10,000 rpm for 10 min. The supernatants obtained were discarded and the pellets were sent to the Servei de Genòmica i Bioinformàtica of the Universitat Autònoma de Barcelona (Bellaterra, Cerdanyola del Vallés, Spain) for 16S rRNA gene sequencing.

#### 2.8.1. Library Preparation

Metagenomics studies were performed by analyzing the variable regions V3–V4 of the prokaryotic 16S ribosomal ribonucleic acid (rRNA) gene sequences, which gave 460 bp amplicons in a two-round PCR protocol.

In the first step, PCR was used to amplify a template out of a DNA sample using specific primers with overhang adapters attached that flank regions of interest. The full-length primer sequences, using standard International Union of Pure and Applied Chemistry (IUPAC) nucleotide codes to follow the protocol targeting this region, were: forward primer: 5′0TCGTCGGCAGCGTCAGATGTGTATAAGAGACAGCCTACGGGNGGCWGCAG and reverse primer: 5′0GTCTCGTGGGCTCGGAGATGTGTATAAGAGACAGGACTACHVGGGTATCTAATCC. PCR was performed in a thermal cycler using the following conditions: 95 °C for 3 min, 25 cycles of (95 °C for 30 s, 55 °C for 30 s, and 72 °C for 30 s), and 72 °C for 5 min. To verify that the specific primers had been correctly attached to the samples, 1 μL of the PCR product was checked on a Bioanalyzer DNA 1000 chip (Agilent Technologies, Santa Clara, CA, USA). The expected size on the Bioanalyzer was ~550 bp.

In the second step, during a limited-cycle PCR, sequencing adapters and dual-index barcodes were added to the amplicon using the Nextera^®^ XT DNA Index Kit, FC-131-1002 (Illumina, San Diego, CA, USA), which allows up to 96 libraries to be pooled together for sequencing on the MiSeq sequencer with the MiSeq^®^ Reagent Kit v3 (600 cycles). PCR was performed in a thermal cycler using the following conditions: 95 °C for 3 min, eight cycles of (95 °C for 30 s, 55 °C for 30 s, and 72 °C for 30 s), and 72 °C for 5 min. Subsequently, the index PCR was run to validate the library by a second Bioanalyzer DNA 1000 chip. The expected size was ~630 bp.

Subsequently, the libraries were quantified using a fluorometric assay and the samples were diluted before pooling. Finally, paired-end sequencing was performed on a MiSeq platform (Illumina) with a 500-cycle MiSeq run (16S Metagenomic Sequencing Library Preparation) [19] using 8 pM samples and a minimum of 20% PhiX. The mean reads obtained were 164,387. Only samples with more than 40,000 reads were used for further analysis. All the sequencing data were deposited by the authors in the Sequence Read Archive and the accession key has been included in the text (PRJNA623853).

#### 2.8.2. Analysis and Processing

The Illumina Basespace 16S Metagenomics app was used for processing and analysis. It performs taxonomic classification of 16S rRNA targeted amplicon reads using a taxonomic database. The app provides interactive visualizations and raw classification output for per-sample and aggregate analyses.

Classification was performed using the Illumina 16S Metagenomics workflow. The algorithm is a high-performance implementation of the Ribosomal Database Project (RDP) Classifier described in a previous study [20]. The RefSeq RDP 16S v3 database is based on FASTA [21].

#### 2.8.3. Richness and Evenness

Richness was defined as the total number of species. Alpha diversity was assessed using the Shannon and Simpson indices. The biodiversity calculations were carried out using the community ecology package (vegan) from R software.

## 3. Results

### 3.1. Analysis of Membranes of Different Materials Using Samples with Known Concentrations of Legionella spp. and Interfering Microorganisms

**Figure 1:**
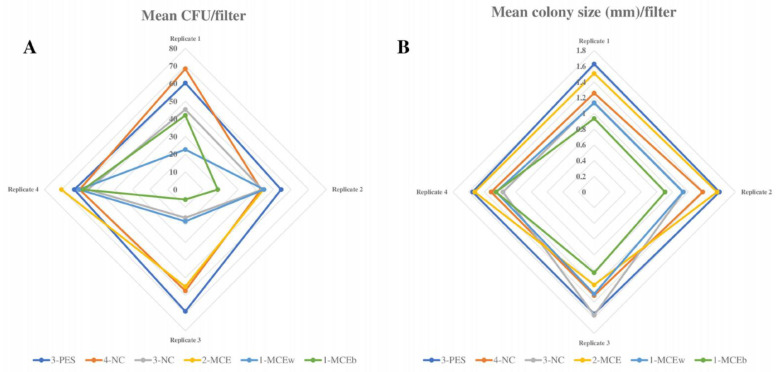
*L. pneumophila* growth on different filtration membranes. (**A**) Radial graph showing mean *L. pneumophila* CFU count obtained with all filtration membranes (results of 4 replicates). (**B**) Radial graph showing mean *L. pneumophila* colony size (mm) obtained with all filtration membranes (results of 4 replicates).

**Figure 2:**
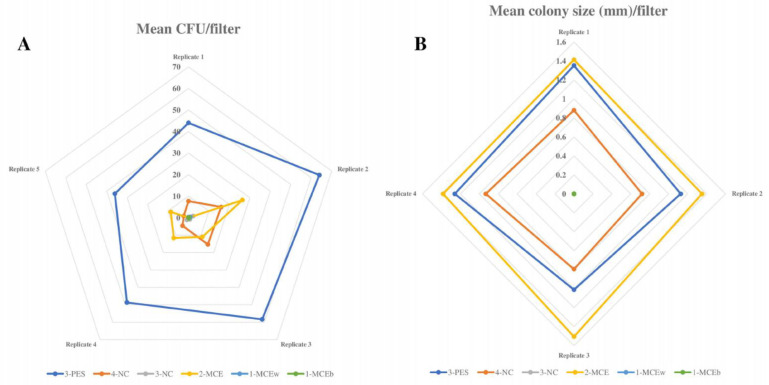
*L. anisa* growth with different filtration membranes. (**A**) Radial graph showing mean *L. anisa* CFU count obtained with all filtration membranes (results of 5 replicates). (**B**) Radial graph showing mean *L. anisa* colony size (mm) obtained with all filtration membranes (results of 4 replicates).

A comparison of filtration membranes of different materials and manufacturers was performed to assess the influence of the filter material on *Legionella* recovery when seeded in the selective medium. Statistical analysis revealed significant differences between groups of filters for the recovery of both *L. pneumophila* and *L. anisa* strains. In particular, 3-PES, 4-NC, and 2-MCE gave significantly higher mean CFU counts and colony sizes than 3-NC, 1-MCEw, and 1-MCEb (Figure 1 and Figure 2).

#### 3.1.1. Productivity Results of High-Recovery Membranes

**Figure 3:**
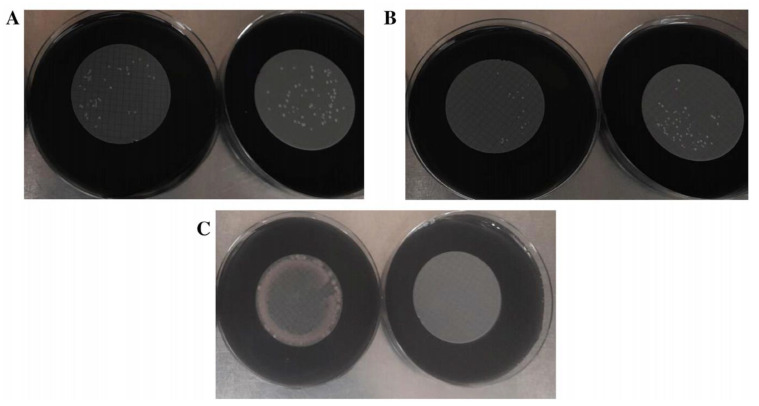
Comparison of microbial growth on 4-NC (left) and 3-PES filters (right). (**A**) *L. pneumophila* growth. (**B**) *L. anisa* growth. (**C**) *P. aeruginosa* growth.

For *L. pneumophila* growth, 3-PES provided the highest mean CFU counts, the differences being statistically significant when compared to 2-MCE (52.27 ± 20.99 CFU vs. 46 ± 22.86 CFU; *p* = 0.027) and without significance in relation to 4-NC (52.27 ± 20.99 CFU vs. 48.27 ± 21 CFU; *p* = 0.143) (Figure 1A and Figure 3A). The same pattern was found for colony sizes, which were significantly larger when using 3-PES vs. 4-NC (1.58 ± 0.15 mm vs. 1.32 ± 0.13 CFU; *p* < 0.001) or 2-MCE (1.58 ± 0.15 mm vs. 1.40 ± 0.28 CFU; *p* = 0.027) (Figure 1B).

In *L. anisa* recovery, the differences between 3-PES and the other materials were more marked: 50.20 ± 12.47 CFU for 3-PES vs. 9.20 ± 6.89 CFU for 4-NC (*p* < 0.001) and 14.42 ± 8.58 CFU for 2-MCE (*p* < 0.001) (Figure 2A and Figure 3B). In the comparison of mean colony sizes, 2-MCE provided larger colonies than 3-PES (1.42 ± 0.17 mm vs. 1.09 ± 0.10 mm; *p* < 0.001) and 4-NC (1.42 ± 0.17 mm vs. 0.79 ± 0.14 mm; *p* < 0.001), while use of 3-PES resulted in significantly larger colonies compared to 4-NC (1.09 ± 0.10 mm vs. 0.79 ± 0.14 mm; *p* < 0.001) (Figure 2B).

#### 3.1.2. Productivity Results of Low-Recovery Membranes

When comparing *L. pneumophila* growth provided by low-recovery membranes (3-NC, 1-MCEw, and 1-MCEb), the highest mean CFU counts were provided by 3-NC, which differed significantly from counts of 1-MCEb (34.20 ± 18.94 CFU vs. 27.07 ± 21.42 mm; *p* < 0.001) but not 1-MCEw (34.20 ± 18.94 CFU vs. 30.93 ± 19.97 CFU; *p* = 0.114) (Figure 1A). The same pattern was observed for the mean colony size of *L. pneumophila*: 3-NC provided larger colonies, with significant differences vs. 1-MCEb (1.27 ± 0.36 mm vs. 0.98 ± 0.14 mm; *p* < 0.001) and without significant differences vs. 1-MCEw (1.27 ± 0.36 mm vs. 1.19 ± 0.15 mm; *p* = 0.424) (Figure 1B).

For *L. anisa*, a partial or total absence of growth was observed with all three filters. The mean CFU numbers obtained with 3-NC were significantly higher compared to counts of 1-MCEw (1.00 ± 1.36 CFU vs. 0.20 ± 0.56 CFU; *p* = 0.011) and 1-MCEb (1.00 ± 1.36 CFU vs. 0.13 ± 0.35 CFU; *p* = 0.008) (Figure 2A). Regarding the mean colony sizes, statistical analyses could not be performed due to the lack of growth of *L. anisa* (Figure 2B).

#### 3.1.3. Selectivity Analysis

Regarding the selectivity properties, all the membranes placed on GVPC agar totally inhibited *E. coli* and *E. faecalis* ATCC 19433 and ATCC 29212, but only 3-PES completely inhibited *P. aeruginosa* (Figure 3C). An accurate quantitative analysis of *P. aeruginosa* growth with the other membranes was not possible due to the presence of high biomass in all samples.

### 3.2. Comparison of Different Polyethersulfone Filters

Three PES membranes of three different brands (2-PES, 3-PES, and 5-PES) were evaluated with GVPC agar to compare their performance in terms of *Legionella* recovery and medium selectivity properties.

For *L. pneumophila*, 3-PES and 2-PES provided significantly higher CFU counts in comparison with NC (109 ± 13.24 CFU vs. 85.4 ± 4.27 CFU; *p* = 0.012 and 115 ± 5.38 CFU vs. 85.4 ± 4.27 CFU; *p* < 0.01), whereas 5-PES resulted in a lower level of growth in comparison with NC (54 ± 20.19 CFU vs. 85.4 ± 4.27 CFU; *p* = 0.027) (Table 2).

In the case of *L. anisa*, the highest recoveries were also obtained with 3-PES and 2-PES, which differed significantly from counts in NC (201.4 ± 22.57 CFU vs. 144.6 ± 19.71 CFU; *p* = 0.002 and 241.8 ± 40.51 CFU vs. 144.6 ± 19.71 CFU; *p* = 0.002). The CFU values obtained with 5-PES and NC were comparable (158.4 ± 9.71 CFU vs. 144.6 ± 19.71 CFU; *p* = 0.209) (Table 2).

**Figure 4:**
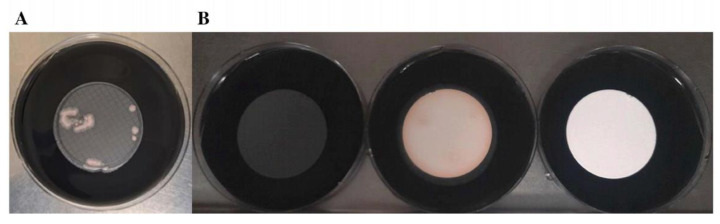
Comparison of *P. aeruginosa* growth with different filters. (**A**) 4-NC as control membrane. (**B**) 3-PES (**left**), 2-PES (**center**), and 5-PES (**right**) as target membranes.

Regarding selectivity properties, all the membranes completely inhibited the growth of strains *E. coli* ATCC 25922, *E. faecalis* ATCC 29212, and *E. faecalis* ATCC 19433. For *P. aeruginosa* ATCC 9027, total inhibition was observed with 3-PES and 5-PES, whereas 2-PES resulted in a high growth. The NC membrane provided some inhibition of *P. aeruginosa*, but not total (1–4 CFU/plate) (Figure 4).

### 3.3. Comparison of Different Membrane Materials in Real Water Samples

#### 3.3.1. Analysis of Water Samples with a Low Concentration of Interfering Microorganisms

Based on the above results, the membranes with higher productivity and selectivity properties (2-MCE, 4-NC, and 3-PES) were assessed in real water samples. Eight different water samples presumed to contain a low concentration of interfering microorganisms (LIM 1–8) due to their origin (water systems) were microbiologically evaluated to confirm the influence of the different membrane materials on the growth rate of *Legionella* spp. and the inhibition properties of GVPC agar.

**Figure 5:**
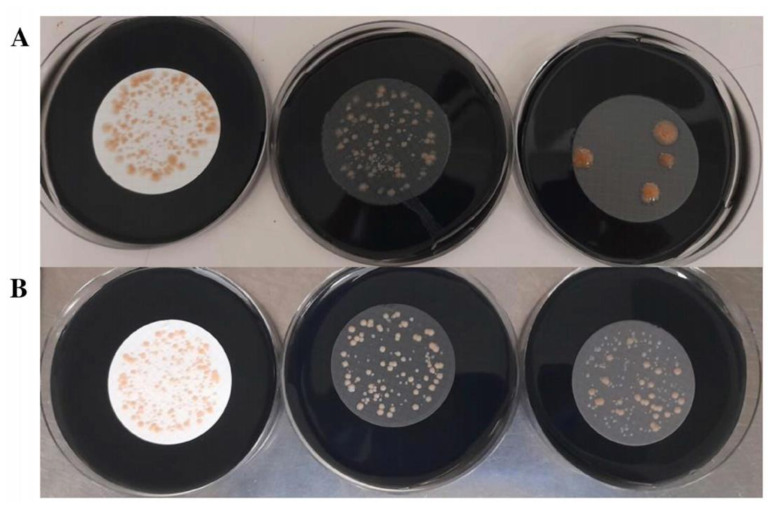
Comparison of different membranes using a real water sample with a low concentration of interfering microorganisms (LIM sample n°3): 2-MCE (**left**), 4-NC (**center**), and 3-PES (**right**). (**A**) Analysis of the pure sample. (**B**) Analysis of the sample inoculated with 500–1000 CFU/L of *L. pneumophila* and 500–1000 CFU/L of *L. anisa*.

In the non-contaminated samples, 2-MCE and 4-NC resulted in lower microbial inhibition than 3-PES (Figure 5A). *Legionella* colonies were not detected in any samples. When analyzing water samples previously inoculated with *Legionella*, a higher recovery was obtained with 3-PES (Figure 5B and Table 3); the mean CFU counts were 45.1 for 3-PES, 32.6 for 2-MCE, and 29.0 for 4-NC. Furthermore, a more accurate *Legionella* CFU count was obtained with 3-PES in comparison with the other two materials, as the medium selectivity was higher when using this filter.

#### 3.3.2. Analysis of Water Samples with a High Concentration of Interfering Microorganisms

Five water samples presumed to have a high microbial concentration (HIM) (HIM 9–13) due to their origin were also microbiologically evaluated to assess the influence of the different membrane materials on the growth of *Legionella* spp. and interfering microorganisms. These samples were submitted to physicochemical treatments (thermal and acid) according to ISO 11731:2017 [9].

**Figure 6:**
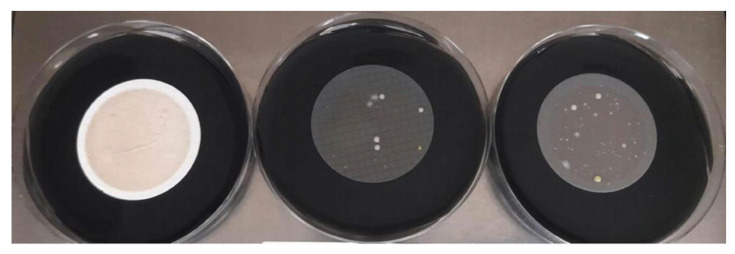
Comparison of different membranes using a real water sample with a high concentration of interfering microorganisms (HIM sample n°4), inoculated with *Legionella* spp., and pre-treated with acidification: 2-MCE (**left**), 4-NC (**center**), and 3-PES (**right**).

The 3-PES membrane was associated with a higher *Legionella* recovery and inhibition of interfering microorganisms. After the thermal and acid treatments, fewer interfering microorganisms were able to grow when 3-PES filters were used compared to those of the other materials (Figure 6 and Table 4). Moreover, 3-PES provided the highest number of CFU for both *Legionella* species, followed by 4-NC (Table 3). The lowest CFU count was obtained with 2-MCE membranes, whose white color also hindered the recognition of *Legionella* colonies.

#### 3.3.3. Results of 16S Analysis

Four of the eight evaluated water samples with a low microbial concentration (LIM samples 5–8) and the five water samples with a high microbial concentration (HIM samples 9–13) were analyzed using metaGenomeSeq.

**Figure 7:**
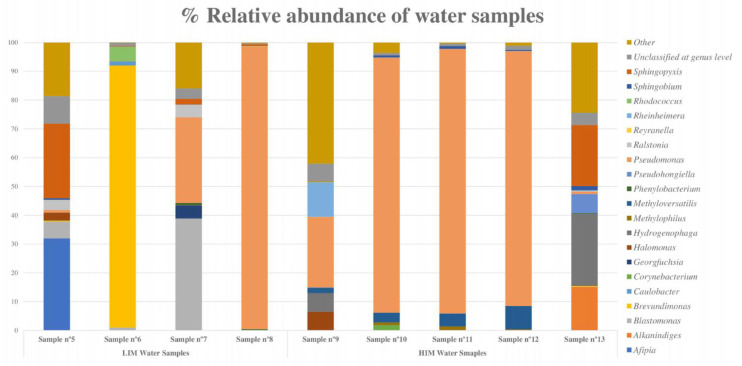
Bar graph showing the percentage of relative abundance of the eight most abundant bacteria in each water sample (LIM and HIM). The “other” category is the sum of all genera with less than 3.50% abundance.

The percentage of genus abundance in the LIM samples indicated a high microbial diversity, particularly in samples 5 and 7. A predominant microorganism was detected in samples 6 and 8: *Brevundimonas* in sample 6, with 89.97% of relative abundance, and *Pseudomonas* in sample 8, with more than 98.20% of relative abundance (Figure 7).

In HIM samples, the metagenomic analysis revealed that *Pseudomonas* was the most abundant genus, with more than 85% relative abundance in samples 10, 11, and 12 and 23.05% in sample 9, followed by *Hydrogenophaga*, which had a high relative abundance in samples 9 and 13 (Figure 7).

**Figure 8:**
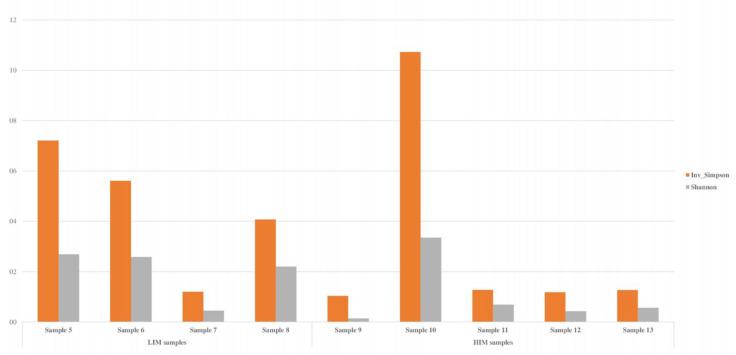
Biodiversity index (Shannon and Simpson indices) of the water samples determined by metaGenomeSeq analysis. These results were calculated using the richness in all samples (*n* = 1017).

The biodiversity index was calculated considering the global richness and evenness, with 1017 bacterial genera detected in the total samples. The results indicated high microbial diversity in samples 5, 6, 8, and 10, according to Shannon’s and Simpson’s indices (Figure 8).

## 4. Discussion

In laboratories performing microbiological analyses of water samples, it is necessary to confirm the required performance of the selected filters, as a prerequisite for any reliable microbiological work [22]

The type of membrane used in filtration has a considerable impact on the recovery of *Legionella* spp. [11,23], which can complicate the interpretation of results and comparison with data from other laboratories. Membrane filtration is recommended for *Legionella* detection in water with low concentrations of bacteria [9], which requires optimal recovery and effective inhibition of interfering microorganisms. In this context, the aim of the present study was to identify the most efficient filtration membrane in terms of *Legionella* growth by comparing filters of different brands made of materials allowed by ISO 11731:2017 for *Legionella* testing [9].

We observed that *Legionella* growth can oscillate depending on the membrane used, with 3-PES and 2-MCE allowing higher growth (CFU numbers) of *L. pneumophila* and *L. anisa* than the other materials. Another notable finding was the high growth of *L. anisa* obtained with the 3-PES membrane; conversely, this species was unable to grow after filtration with NC and MCE (1-MCEw, 1-MCEb, and 3-NC). Although good productivity was also obtained with 2-MCE and 4-NC, it was significantly lower compared to that of 3-PES. High recovery rates of *L. anisa* strains are essential in water sample analysis, as this species requires longer incubation periods than *L. pneumophila*, which hinders detection [24]. The detection of *L. anisa* in GVPC is also important because it is the most common non-*pneumophila Legionella* species in the environment, is a causative agent of legionellosis and Pontiac fever, and can be hospital-acquired [25,26]. Epidemiological studies have found that the homes of approximately 20% of Legionnaires’ disease patients tested positive for *Legionella*, mostly *L. anisa* [27,28].

As expected, the same pattern was observed when testing real water samples after filtration, with PES providing higher productivity. Similar results were obtained in previous studies performed by a PES filter manufacturing company [29], which reported that black PES membranes allowed significantly higher *L. pneumophila* and *L. anisa* growth in comparison with MCE and NC filters. Additionally, as well as a better recovery, a more homogeneous morphology of colonies was obtained [29].

The enhanced growth of *Legionella* spp. on PES membranes may be attributed to the pore size distribution. According to several studies on dialysis membranes, PES is highly suitable for the manufacture of a range of membranes, having the advantage of a narrow pore size distribution, which confers higher selectivity [14,30], in contrast with materials such as cellulose, which have a broader pore size distribution. Bacterial growth can also be affected by pore size and an improved pore distribution may facilitate the transport of components essential for *Legionella* growth from the culture medium, although this requires further investigation.

Having determined the superiority of PES as a filter material, PES membranes from three different brands were compared. Although they all provided a high *Legionella* recovery, statistically significant differences were observed among them. As in the previous assays, the best results were obtained with 3-PES, with the other two membranes performing less well in terms of productivity or selectivity. The differences in growth obtained with the different PES membranes could be related to variables such as pore size, electrostatic charges, and content of inhibitory substances [31,32]. Porous polymeric membranes can be made using several techniques, such as non-solvent induced phase separation, vapor-induced phase separation, electrospinning, track etching, and sintering, and the processing parameters of each method (for example, the type and composition of solvent and non-solvent systems, or the composition and concentration of the polymer solution) influence filter morphology and performance [33].

It is known that properties of filters can influence the retention of microorganisms and the diffusion of nutrients and macromolecules such as antibiotics [34], with the differences in surface chemistry and pore morphology being relevant.

In the characteristics of different filters and brands, we have noted that 3-PES is the thinnest filter, at 130 µm (while NC and MCE can be up to 152 µm thick). This could imply a higher availability of essential nutrients, such as Fe^3+^ and cysteine, explaining the highest *Legionella* growth observed in 3-PES. In fact, according to ISO 7704 [35], membrane filter counts represent 80 to 90% of those obtained by plate counts using the same culture medium, thus highlighting that diffusion of nutrients is not always complete.

Another significant finding of the present study is that the membrane material can enhance the selectivity properties of GVPC agar. All filters provided total inhibition of *E. faecalis* (ATCC 29212 and ATCC 19433) and *E. coli*, but *P. aeruginosa* growth was only inhibited when using PES filters. The same microbiological pattern was observed in the real water samples, with the highest selectivity being achieved with PES membranes.

Among the different filters, there are differences in bubble point values (23 psi for 3-PES), which is an indirect measure of pore size, wettability, surface tension, and angle of contact.

The superficial characteristics could have great influence in the selectivity differences observed, which could be attributed to a differential interaction between the antibiotics (polymyxin and vancomycin) and filter. According to a recent study, the properties of filters can influence the diffusion of macromolecules such as antibiotics [34].

In additional non-published studies performed by our group, we have compared the diffusion of the antibiotics polymyxin and vancomycin through the filters before the filtration of the bacterial suspension and its effects on the selectivity properties of the medium against *P. aeruginosa* and other interfering microorganisms. Preliminary results are showing relevant differences among the different materials, in which PES could allow higher diffusion of polymyxin, maybe due to pore distribution and lower thickness. This effect could explain the results obtained in our study, in which 3-PES provided particular selectivity against *P. aeruginosa* and other interfering microorganisms present in real water samples.

Furthermore, in a previous study, we reported that the non-inhibition of *P. aeruginosa* on GVPC agar may be partly attributed to the interaction between activated charcoal and polymyxin B, which can inactivate the antibiotic effect [10]. By modifying the charcoal and by preparing the medium in the absence of oxygen, this interaction can be reduced [10]. It is possible that both factors, increased polymyxin availability in the medium and PES properties, allowing more diffusion of polymyxin, would increase the selectivity properties against *P. aeruginosa*. According to the present results, the use of PES filters with GVPC agar prepared in the absence of oxygen constitutes an effective method for *P. aeruginosa* inhibition in water samples.

The water samples were also analyzed by 16S metagenomic sequencing to determine their bacterial composition. The predominant species identified were Gram-negative bacteria commonly found in water systems, such as *Pseudomonas, Hydrogenophaga, Brevundimonas, Afipia*, and *Sphingopixis* [36,37,38,39]. *Pseudomonas* was particularly abundant in samples with a high microbial content.

In conclusion, the results of this study support the use of PES membranes for the detection of *Legionella* spp. in water samples. We have demonstrated that the filter membrane material can strongly influence the growth of *Legionella* spp. (particularly *L. anisa*) and the inhibition of interfering microorganisms. Specifically, PES membranes exhibited higher productivity and selectivity than those made of other materials such as NC or MCEs. Thus, we suggest that for *Legionella* detection in water samples, the use of PES filters should not be restricted to procedures where membrane filtration is followed by a washing step, as stipulated in ISO 11731:2017 [9], but could be extended to processes in which the membrane filter is placed directly on the culture media.

## Figures and Tables

**Table 1:** List of filtration membranes evaluated in this study, classified by brand, membrane material, color, and pore size.

Abbreviations	Manufacturer	Material	Color	Pore Size (mm)
1-MCEW	1	Mixed cellulose esters	White	0.45
1-MCEB	1	Mixed cellulose esters	Black	0.45
2-MCE	2	Mixed cellulose esters	White	0.45
2-PES	3	Hydrophilic modified polyethersulfone	White	0.45
3-NC	3	Nitrocellulose	White	0.45
3-PES	3	Hydrophilic modified polyethersulfone	Grey	0.45
4-NC	4	Nitrocellulose	Grey	0.45
5-PES	5	Hydrophilic modified polyethersulfone	White	0.45

**Table 2:** *Legionella* growth on polyethersulfone (PES) membranes. CFU count of samples containing *Legionella* spp. or *P. aeruginosa* strains seeded on PES membranes. Statistical analysis of *L. pneumophila* recovery with the membranes. Statistical analysis of *L. anisa* recovery with the PES membranes. *p*-values lower than 0.05 indicate statistically significant differences.

Mean CFU Target Strains	Membranes			
	4-NC (Control)	2-PES	3-PES	5-PES
*L. pneumophila*	85.4 ± 4.27	115 ± 5.38	109 ± 13.24	54 ± 20.19
*L. anisa*	144.6 ± 19.71	241.8 ± 40.51	201.4 ± 22.57	158.4 ± 9.71
*P. aeruginosa*	4	>1000	0	0
***p*** **-value** ***L. pneumophila***	**Membranes**			
	**4-NC (Control)**	**2-** **PES**	**3-** **PES**	**5-** **PES**
4-NC (Control)	-	< 0.01	0.012	0.027
2-PES	<0.01	-	0.391	<0.01
3-PES	0.012	0.391	1	<0.01
5-PES	0.027	<0.01	<0.01	-
***p*** **-value** ***L. anisa***	**Membranes**			
	**4-NC (Control)**	**2-** **PES**	**3-** **PES**	**5-** **PES**
4-NC (Control)	-	<0.01	<0.01	0.209
2-PES	<0.01	-	0.099	0.011
3-PES	<0.01	0.099	-	0.011
5-PES	0.209	0.011	0.011	-

**Table 3:** CFU count of water samples with a low concentration of interfering microorganisms (LIM) filtered with the nitrocellulose (4-NC), mixed cellulose esters (2-MCE), and polyethersulfone (3-PES) membranes. * means the CFU could not be quantified.

LIM Water Samples	Membranes					
	2-MCE		4-NC		3-PES	
	*Legionella*	Interfering Microorganisms	*Legionella*	Interfering Microorganisms	*Legionella*	Interfering Microorganisms
1	*	>100	*	>100	21	0
2	126	0	124	0	229	0
3	13	10	7	10	28	0
4	0	29	0	23	0	25
5	11	0	6	0	20	0
6	13	0	8	0	19	0
7	*	>1000	*	>1000	16	11
8	*	>1000	*	155	4	41

**Table 4:** CFU count of water samples containing a high concentration of interfering microorganisms (HIM) filtered with the nitrocellulose (4-NC), mixed cellulose esters (2-MCE), and polyethersulfone (3-PES) membranes. * means the CFU could not be quantified.

HIM Water Samples	Water Treatment				Membranes		
		2-MCE		4-NC		3-PES	
		*Legionella*	Interfering Microorganisms	*Legionella*	Interfering Microorganisms	*Legionella*	Interfering Microorganisms
9	Untreated	*	>1000	*	>1000	*	>1000
	Temperature	*	>1000	*	>1000	*	>1000
	pH	*	>1000	*	>1000	*	>1000
10	Untreated	0	8	0	10	0	15
	Temperature	0	0	0	0	0	0
	pH	4	2	7	5	7	7
11	Untreated	1	1	0	2	0	1
	Temperature	0	0	0	1	0	2
	pH	3	3	12	1	13	4
12	Untreated	0	2	0	4	0	3
	Temperature	0	0	0	0	0	3
	pH	0	4	7	8	15	14
13	Untreated	*	>1000	*	>1000	*	>1000
	Temperature	*	>1000	*	>1000	*	>100
	pH	*	>1000	*	>1000	*	>100

## Data Availability

Raw data will be available upon request.

## Abbreviations

GVPC	Glycine–Vancomycin–Polymyxin–Cycloheximide
ISO	International Organization for Standardization
PES	Polyethersulfone
MCE	Mixed Cellulose Ester
NC	Nitrocellulose
CFU	Colony-Forming Unit
LIM	Low Concentration of Interfering Microorganisms
HIM	High Concentration of Interfering Microorganisms
